# DNA Damage as a Driver for Growth Delay: Chromosome Instability Syndromes with Intrauterine Growth Retardation

**DOI:** 10.1155/2017/8193892

**Published:** 2017-11-12

**Authors:** Benilde García-de Teresa, Mariana Hernández-Gómez, Sara Frías

**Affiliations:** ^1^Laboratorio de Citogenética, Instituto Nacional de Pediatría, Mexico City, Mexico; ^2^Programa de Doctorado en Ciencias Biomédicas, UNAM, Mexico City, Mexico; ^3^Universidad Anáhuac, Mexico City, Mexico; ^4^Departamento de Genética, Instituto Nacional de Perinatología, Mexico City, Mexico; ^5^Instituto de Investigaciones Biomédicas, UNAM, Mexico City, Mexico

## Abstract

DNA is constantly exposed to endogenous and exogenous mutagenic stimuli that are capable of producing diverse lesions. In order to protect the integrity of the genetic material, a wide array of DNA repair systems that can target each specific lesion has evolved. Despite the availability of several repair pathways, a common general program known as the DNA damage response (DDR) is stimulated to promote lesion detection, signaling, and repair in order to maintain genetic integrity. The genes that participate in these pathways are subject to mutation; a loss in their function would result in impaired DNA repair and genomic instability. When the DDR is constitutionally altered, every cell of the organism, starting from development, will show DNA damage and subsequent genomic instability. The cellular response to this is either uncontrolled proliferation and cell cycle deregulation that ensues overgrowth, or apoptosis and senescence that result in tissue hypoplasia. These diverging growth abnormalities can clinically translate as cancer or growth retardation; both features can be found in chromosome instability syndromes (CIS). The analysis of the clinical, cellular, and molecular phenotypes of CIS with intrauterine growth retardation allows inferring that replication alteration is their unifying feature.

## 1. DNA and Genomic Integrity

DNA is our genetic heritage; the genetic instructions that cells use to construct their components and function are encoded in their sequence. DNA is also the molecule responsible for transmitting information from generation to generation on a cell and organism scale. This information is provided to each human being in the nucleus of the fertilized egg in a set of 46 DNA molecules forming chromosomes. Subsequent divisions generate millions of cells to form a fetus: each one of these cells has its own set of 46 chromosomes. Amazingly, in spite of having been copied millions of times, the DNA sequence of these chromosomes is remarkably similar to the original molecule. This is surprising since DNA is constantly exposed to endogenous and exogenous mutagenic stimuli. On the one hand, each replication round can result in thousands of lesions while, on the other hand, environmental genotoxic agents are a constant and an inevitable source of DNA damage [1, 2]. Every day, the DNA of a fetus can then accumulate tens of thousands of lesions that could result in mutations [2]. However, the DNA molecule is such an important asset to the cell that a significant share of its genetic information and cellular energy are destined to the detection and repair of DNA damage to preserve the organism's genetic integrity.

## 2. DNA Damage and the DNA Damage Response

**Figure 1:**
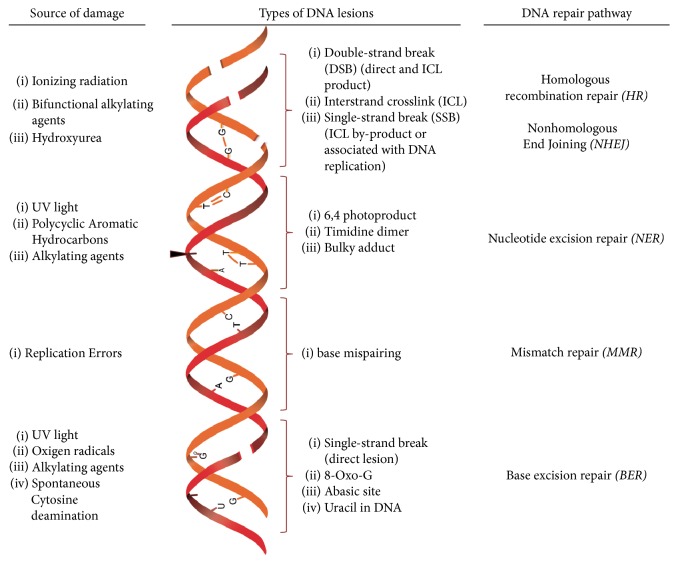
DNA lesions are induced by endogenous and exogenous sources. Every agent causes a particular lesion that will activate specific DNA repair pathways.

The sources of induced lesions in DNA, can be endogenous or exogenous. The former originate from normal metabolic processes inside the cell, such as DNA replication, which may incorporate noncomplementary Watson–Crick bases during DNA synthesis. It can also come from lesions caused by oxidative damage that occurs during normal metabolism in the mitochondria and other cellular sites, giving rise to the oxidation of several cellular components, including DNA, resulting in modified bases or breaking of the union between them. Besides, spontaneous decay of the DNA molecule may generate hydrolysis that creates abasic sites and deamination, causing a change in the original bases. Exogenous sources that continually damage our DNA may be of four main origins: (1) biological, such as some virus, (2) physical, like solar radiation or radiation therapy, (3) chemical, like pesticides and medical treatments (chemotherapy), and (4) personal habits, such as smoking (Figure 1). All together, these mechanisms may generate more than ten thousand lesions per cell per day [3].

Two main factors, the type of DNA lesions and the phase of the cell cycle where they are sensed, affect the choice of the DNA repair pathway to be used and the subsequent outcome (Figure 1). Mispaired bases are repaired by mismatch repair (MMR); oxidative damage, abasic sites, and uracil in DNA are corrected by removing the altered base through base excision repair (BER); UV radiation damage and bulky adducts that disrupt the structure of the double helix are repaired by nucleotide excision repair (NER), in which an oligonucleotide of 30 bp containing the lesion is removed [4]. Most DNA lesions interfere with DNA replication and transcription, processes that are indispensable for appropriate cell function. However, double-strand breaks (DSBs), in which both DNA strands lose continuity, are the most dangerous type of DNA damage and have primarily cytotoxic or cytostatic consequences [2, 3, 5].

Interstrand crosslinks (ICLs) are detected and removed through the Fanconi anemia pathway [6, 7], also known as the FA/BRCA pathway. The processing of ICLs occurs during the S phase, resulting in the following intermediary lesions: an adduct and a DSB, which, are further taken care of by known repair pathways. The adduct is repaired by the NER pathway, while the DSB is processed by one of four independent pathways: nonhomologous end joining (NHEJ), homologous recombination (HR), alternative-NHEJ (alt-NHEJ), and single-strand annealing (SSA). The selection of the repair pathway that will take care of the DSB depends on the cell cycle phase and if the 3′ ends of the DSBs are processed by an exonuclease or not [8]. NHEJ operates in any phase of the cell cycle and does not require any processing since this repair pathway marks both blunt ends of the DSB with a Ku70/Ku80 heterodimer and joins them. Meanwhile, in order to repair a DSB using HR, alt-NHEJ, or SSA, the 3′ blunt ends must be processed to allow the loading of the MRN complex (a heterotrimer integrated by three proteins called MRE11, RAD50, and NBN) to continue the repair process. This occurs primarily during the S and G2 phases of the cell cycle, when sister chromatids are available and can be used for HR error-free repair [7, 9].

## 3. DNA Damage Response

Even when each specific DNA lesion stimulates a particular DNA repair mechanism, cells trigger a common general program known as the DNA damage response (DDR) which is charged with lesion detection, signaling, and repair promotion as well as cell cycle progression control. It is no surprise that the DDR is an extremely controlled process; the exquisite balance between cell survival and cell death and senescence relies on it [1, 9, 10]. Tissue homeostasis or growth abnormalities such as cancer [11] or tissue hypoplasia depend, among other things, on the amount and type of genomic damage sensed and processed by the DDR.

**Figure 2:**
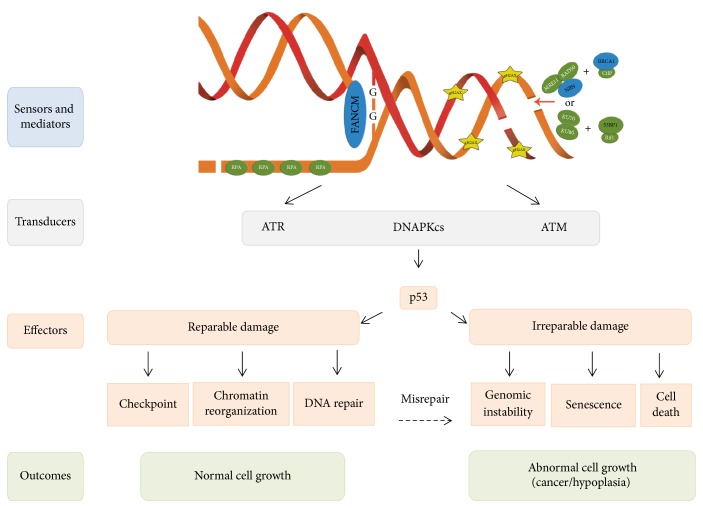
DNA damage response can result in different outcomes following single strand breaks, interstrand crosslinks and double strand breaks. Each DNA lesion is recognized by specific sensor proteins according to the cell cycle phase in which the cell is; during S phase, the protein FANCM identifies a replication fork arrested by an ICL, MRN + BRCA1 sense DSBs; meanwhile, Ku70/Ku80 + 53BP1 can recognize DSBs across the entire interphase; RPA detects and covers ssDNA primarily during S phase. The sensing process is then transduced and amplified on chromatin through a series of posttranslational histone modifications such as phosphorylation of H2AX (*γ*H2AX) on both sides of the DSB. These changes allow the recruitment of the specialized transducer kinase ATR that mainly responds to RPA-covered ssDNA originated by replication stress or DNA lesions such as ICLs; concurrently, ATM responds to DSBs. Kinases activate several effector proteins including the transcription factor p53, which acts downstream regulating diverse outcomes according to the type and quantity of reminding lesions in the cell.

The DDR is composed of a network of regulatory noncoding RNAs and proteins that act as sensors, transducers, and effectors [1, 12]. Sensor proteins recognize specific lesions (Figure 2). For example, the FANCM protein detects the stalled replication fork caused by interstrand crosslinks (ICLs); the MRN complex is the typical sensor of DSBs during S/G2 phase; the Ku70/Ku80 heterodimer is the primary DSBs sensor during G1, while replication protein A (RPA) overcoats single-strand DNA (ssDNA) found either at processed DSB overhangs to be repaired by HR or at stalled replication forks [13].

Following recognition of the lesions by sensors and mediators, the signal is amplified by transducer proteins. The PIKK kinases (Phosphatidyl Inositol 3-Kinase-related Kinases) family is the most important of this group; it is integrated by ataxia telangiectasia and Rad3-related (ATR), Ataxia telangiectasia-mutated (ATM), and DNA dependent protein kinase (DNAPKcs). ATR is activated during the S phase of the cell cycle, in the presence of DNA damage such as base adducts, crosslinks, single-strand breaks (SSBs), replication stress, and DSBs that originate during the S phase, while ATM and DNAPKcs are activated by DSBs at any point of the cell cycle [13, 14]. The DDR kinases activate signaling cascades through posttranslational modifications of various proteins, including phosphorylation, ubiquitylation, and PARylation, which play a central role in regulation of the DDR [15]. Specifically, ATR and ATM can autoactivate or transactivate each other through phosphorylation and then phosphorylate the histone variant H2AX (*γ*H2AX) that acts as a platform to recruit DDR factors and prepare the cell to restore DNA integrity. ATR and ATM's main downstream phosphorylation targets are proteins Chk1 and Chk2 and the important effector p53; their activation allows regulating (a) the chromatin structure surrounding the lesion, (b) checkpoints that stop the cell cycle, (c) DNA repair proteins, (d) proteins that induce senescence, and (e) proteins that lead to cell death (Figure 2) [10, 16].

Finally, if DNA lesions are repaired, the checkpoint that is responsible for stopping the cell cycle is turned off and the cell cycle is allowed to restart through a process called checkpoint recovery. This process is only turned on when the signaling for the DNA lesions is silenced, and the surviving cell recovers its normal homeostasis and growth. When DNA damage cannot be properly repaired, the cell's destiny should be either senescence or death. Otherwise, cells might divide with unrepaired DNA damage through an erroneous activation of the checkpoint recovery process that allows cell survival with genomic instability to promote cell dysfunction and cancer [17].

## 4. DNA Damage Response and Disease

Unrepaired or misrepaired lesions in DNA may immediately impair replication and transcription, affecting the whole cell function. In an irreparable damage situation, the amount and type of lesions, as well as the cell cycle phase in which the cell is, will influence the DDR response in order to favor immediate cell death or the conversion of DNA lesions into durable mutations or stable chromosomal abnormalities [3].

Cell survival, despite genomic damage, can directly affect cell growth; this can be evidenced by two opposite outcomes: On the one hand, there can be an overgrowth effect, since mutations in critical genes such as oncogenes or tumor suppressor genes can alter cell function and increase the likelihood of cancer development [3, 18, 19]. On the other hand, there can be cell hypoplasia or cell loss; this happens when irreparable damage leads to either cell death or cell senescence, a cancer-protecting condition in which cells are alive but unable to proliferate, limiting the growth of the tissues and conducting to aging (Figure 2). This is evidenced by the phenotype of patients affected by diseases that alter the DDR and DNA repair in which growth alteration and increased cancer susceptibility are important features.

## 5. Chromosome Instability Syndromes

Mutations in at least 114 genes involved in the DDR lead to disease; some of the mutations are somatic, resulting in various types of spontaneous cancer, and others are genetic diseases with constitutional mutations that lead to syndromes that may or may not be related to the development of cancer [10]. Among the genetic diseases with mutations in DDR genes, the chromosomal instability syndromes (CIS) are a group of rare Mendelian diseases, characterized by increased chromosome breakage resulting from unrepaired or misrepaired DNA strands breaks. Other than chromosomal instability, they also have clinical overlapping features like cancer proneness, premature aging, and growth abnormalities, even though each CIS has a particular phenotype that distinguishes it from the others. Only a subgroup of these patients has prenatal growth alterations; this review focuses on those syndromes (Table 1).

## 6. DDR Alteration and Growth Failure

Historically, disruption of the DDR has been associated with cell growth abnormalities in a unilateral way: always pointing towards the increased cell number in the form of deregulated cell proliferation in cancer [19]. But there is little information on how DDR alterations can also lead to deregulation of growth in the opposite direction, resulting in lack of growth due to cell death and low cell reproduction. One of the first observations to partner growth deficiency with an altered DDR was the finding that some Seckel syndrome patients (SS), a rare phenotype consisting of primordial dwarfism and microcephaly, had mutations in the* ATR* gene, which encodes the ATR kinase, a DDR transducer. Further analysis of SS patients revealed locus heterogeneity for this phenotype; mutations in other genes related to DDR or DNA repair also led to this clinical picture. This observation has reinforced the association between DDR failure and severe intrauterine growth retardation. Moreover, genes that participate in centrosomal biology are also related to the uncommon phenotype of microcephalic primordial dwarfism, suggesting that centrosomes have a central role for the adequate differentiation of early neuroprogenitors. It has then been proposed that failure in cell proliferation, secondary to abnormal mitosis, is the cause of primordial dwarfism, since the multipolar spindles result in the activation of checkpoints, reducing proliferation and activating apoptosis [20].

For the phenotype of growth restriction to be evident prenatally, the mechanisms regulating growth must be compromised from the beginning of the development in embryonic stem cells (ESCs) which are pluripotent cells derived from the three primary germ layers: ectoderm, endoderm, and mesoderm, and that have the capacity to differentiate into more than 200 cell types. These cells have been proven to have an extremely efficient DDR and proficient DNA repair mechanisms that allow for a rigorous maintenance of their genome integrity; otherwise, unrepaired DNA damage in ESCs would be amplified, affecting normal human development.

ESCs have an extremely active proliferation rate; their cell cycle has a characteristically reduced G1 phase, and repair mechanisms are heightened. When comparing ESCs to fibroblasts, it stands out that MMR is enhanced, BER and NER repair pathways are highly competent, and the repair of DSBs is preferentially made through the reliable HR pathway [21]. Moreover, when HR is not available and DSBs accumulate, apoptosis is the preferred route to remove highly damaged cells. It is interesting that DSB repair in ESCs requires signaling through ATR instead of ATM. ESCs have a special mechanism to deal with DNA damaged cells when inefficient DNA repair or DDR affects the induction of apoptosis or autophagy [22]. This mechanism leads to ESC differentiation that is driven by damage-induced expression of the p53 transcription factor; damaged differentiated cells are efficient in cell cycle arrest and apoptosis. This strategy results in the maintenance of genetic stability in a decreased number of ESCs and a portion of differentiated cells that become senescent, both resulting in growth deficiency [21].

Growth deficiency is at the center of many diseases, including extremely low body size. In mammals, overall size is determined by the number of cells; body mass is then the sum of all the cells that have proliferated minus those that have died. A fetus that is growing harmonically has accurate balance between these two processes; failure of either one of these results in dramatic effects when they occur in the ESC population; a decrease in cell proliferation rate or an increase in apoptosis leads to intrauterine growth retardation (IUGR) [23].

## 7. Chromosome Instability Syndromes with Intrauterine Growth Retardation

### 7.1. Seckel Syndrome

Seckel syndrome (SS) is an autosomal recessive disorder, clinically and genetically heterogeneous. So far, at least six genes have been associated with the Seckel phenotype; most of these genes participate in ATR-mediated DDR and in centrosomal function. Two clinical subgroups can be identified: on the one hand, patients with mutations in* ATR*,* NIN, *and* ATRIP* genes only present the Seckel phenotype, while, on the other hand, those with mutations in* CENPJ*,* CEP152, or RBBP8 *show allelic heterogeneity and, besides the SS phenotype, can also manifest as a spectrum of disorders known as Primary Autosomal Recessive Microcephalies [24–28]. SS patients who bear mutations in* ATR* show a classical Seckel phenotype, the SS data presented here will be limited to such patients.

*ATR *is localized in 3q23; it is one of the serine threonine kinases that belong to the PIKK family. They have a fundamental role in the DDR since they participate as transducers in the signaling of DNA lesions, especially when the DDR responds to stalled replication forks and bulky lesions in DNA [24, 25]. ATR responds preferentially to ssDNA during the S phase. It also plays a crucial role in preventing DNA breaks caused by fork pausing when DNA replication machinery finds DNA lesions or complex DNA structure and sequences [24]. ATR stability depends on binding of ssDNA to the ATR cofactor, ATRIP (ATR, interacting protein), and the single-stranded binding protein (RPA). ATRIP is required for ATR localization to the ssDNA regions and hence for ATR activation [25, 27]. Major functions of ATR are activation of cell cycle check point arrest, stabilization of stalled replication forks, and promotion of replication fork restart, which is achieved through its ability to phosphorylate a wide range of substrates that include p53 and H2AX [27, 29].

Cellular phenotype of ATR deficient cells is characterized by decreased phosphorylation of ATR-dependent substrates as well as an impaired G2/M checkpoint arrest. These cells are characterized by markers that signal unrepaired DSBs, like the presence of *γ*H2AX, chromosomal breakage, [26, 29, 30] and micronuclei formation. This cellular phenotype may be originated by a failure to recover from replication stalling, which generates DSBs that are normally repaired by HR when they occur during S/G2. In SS cells, the absence of ATR is critical during embryonic development; this deficiency leads to genomic instability, cell senescence, and cell death that leads to fetal growth and accelerated aging [31].

Moreover, it appears that* ATR* and the other SS genes not only participate in the DDR, but also take part in the control of centrosome maturation. This engagement in centrosome biology is also true for other DDR proteins like BRCA2; biallelic mutations in its gene are responsible for a subgroup of Fanconi anemia patients [32]. The unifying feature between all Seckel patients, irrespective of the molecular defect, is that their cells have an altered cell cycle progression that is particularly evident during high rate cell division, when there is hypersensitivity to replicative stress and centrosomal dysfunction. This is especially important in cells with high division rates, like the ones found in developmental stages where rapid replication is key [24, 27].

**Figure 3:**
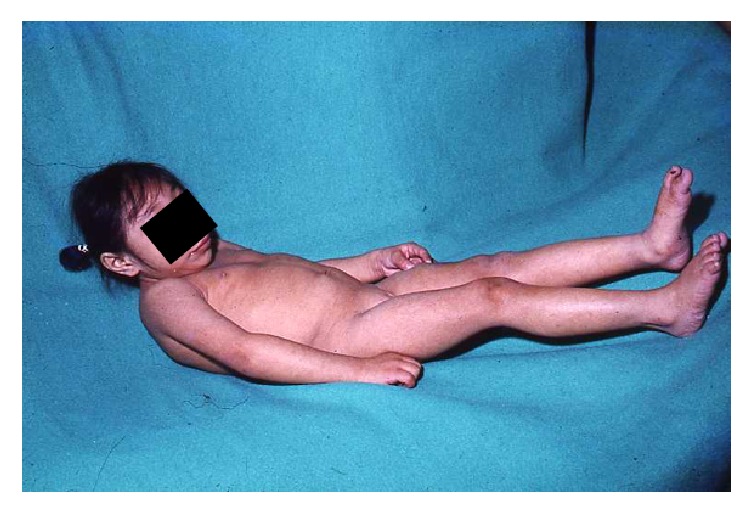
Seckel syndrome patient. Note the severe microcephaly and the “bird-like” appearance facies with micrognathia and receding forehead. The patient is severely disabled with no independent march.

SS is characterized by IUGR, dwarfism, microcephaly (below −4 SD), mental retardation and some other anomalies like luxation of the head of the radius, scoliosis, bone age delay, and seizures (Figure 3). The distinctive facial features include a prominent nose with micrognathia and a receding forehead resulting in a “bird-like appearance” [26, 31, 33]. Since this syndrome has important clinical heterogeneity, other anomalies have been reported, including mandibular hypoplasia, sternal abnormalities, clinodactyly, and low set ears with hypoplastic lobules. Although rare, other reported features are moyamoya syndrome, osteosarcoma, and polyarteritis nodosa [26]. Cerebral malformations, like neuronal migration abnormalities, have been described but are not always present [33]. No glucose metabolism abnormalities have been reported.

Dwarfism can be related to a reduction in the total number of cells generated during development, leading to hypoplastic tissue and reduced organism size [23]. On the other hand, microcephaly may be related to an impaired DNA damage response signal that could alter the cellular threshold for cell death resulting from DNA damage, increasing the levels of apoptosis during development; developing neurons are rapidly proliferating and potentially generate high levels of oxidative damage, which may lead to a higher level of lesions being faced at replication forks [23, 25]. Also an altered mitosis, secondary to impaired spindle formation, could delay mitotic progression and increase the proportion of nonviable cell divisions. Finally, the stem cells may be affected by abnormal centrosomal function. Stems cells have asymmetric divisions in order to preserve the characteristics of a stem cell, and the centrosomes play an important role in such divisions [23].

There is limited clinical information regarding age-related diseases in SS; there is a void of prospective follow-up data from these patients that can only be obtained by following a cohort of SS patients, something that is apparently not being done at this time. Nevertheless, animal models bring some clues over the possibility of an aging phenotype in SS patients since ATR deficient adult mice show premature age-related phenotypes, as well as increased deterioration of tissue homeostasis [29, 34].

### 7.2. Fanconi Anemia

**Figure 4:**
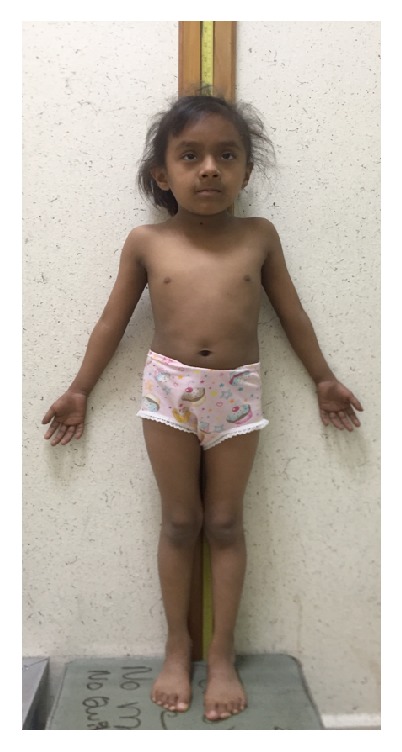
Fanconi anemia patient. This six-year-old girl is 105 cm tall; her height is just below the 5th percentile, and her at-term birth weight is reported to be of 1900 gr. Characteristic features of FA are evident: skin hyperpigmentation, bilateral radial defects consisting of bilateral thenar hypoplasia, and absent fold of the right thumb which cannot be bent.

DNA interstrand crosslinks are extremely noxious DNA lesions that affect central cellular processes like transcription and replication. The FA/BRCA pathway is responsible for the appropriate processing of these lesions; protein products from at least 21* FANC* genes participate in this pathway [35]. The malfunction of any of those FANC proteins leads to the clinical phenotype known as Fanconi anemia (FA), which is characterized by short stature, congenital malformations from the VACTERL-H spectrum, bone marrow failure, and an increased susceptibility to cancer like acute myeloid leukemia, epithelial head, and neck cancer [36] (Figure 4). Most of the FA families bear biallelic mutations in* FANC* genes that have an autosomic recessive inheritance pattern; only families with mutations in* FANCB* show an X-linked recessive pattern, while those in which* FANCR* is the affected gene demonstrate an autosomic dominant one [35].

The FA/BRCA is an S-specific pathway that has 3 basic steps: it starts with the detection of the ICL by the protein FANCM, followed by the recruitment of the FA core complex that is responsible for the monoubiquitination of the FANCD2/I heterodimer which, in turn, favors the recruitment of effector FANC-repair proteins that restore the DNA to its original form [35].

**Figure 5:**
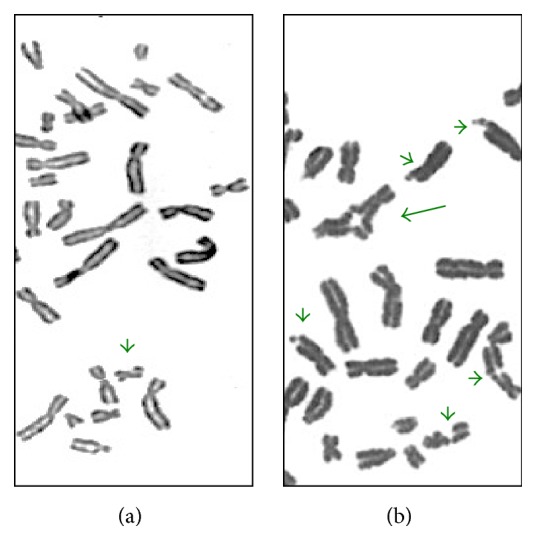
Chromosomal instability induced by 40 ng/ml mitomycin C. (a) Lymphocytes from a healthy individual; (b) lymphocytes from a FA patient. Short arrows show chromosomal breaks; the long arrow shows a quadriradial figure. Note the exacerbated chromosome instability found in the FA patient.

The cellular phenotype of FA derived cells is extremely constant. An aberrant FA/BRCA pathway can result in unrepaired DSBs that manifest as chromosomal breaks, or abnormal repair by an error-prone repair pathway that results in radial figures. These alterations are cytogenetic evidence of chromosomal instability, which is the hallmark of FA cells and is exploited for clinical diagnostic purposes (Figure 5). The agents capable of inducing this chromosomal instability are from endogenous origin, like aldehydes [37] and reactive species of oxygen (ROS) [38], as well as exogenous sources like bifunctional alkylating agents (diepoxybutane or mitomycin C). In addition, FA cells have accumulation of cells in G2 phase of the cell cycle, resulting from a functional G2/M checkpoint and a proapoptotic phenotype.

In contrast with the cellular FA phenotype, the clinical picture in FA patients is extremely variable; not every patient will have all the manifestations: one-third of patients do not have congenital malformations [39], while almost 90% will develop bone marrow failure [36]. One of the more constant manifestations in FA patients is growth parameters alteration. An analysis from the international Fanconi anemia registry (IFAR) data showed that over 60% of FA patients presented short stature (below the 5th percentile), while birth length and weight below the 5th percentile was respectively reported in almost 30% and nearly half of FA patients [40]. Moreover, a prospective study of data from 54 IFAR participants showed that the mean standard deviation score (SDS) for height in these patients was significantly below normal for age and sex at −2.35 ± 0.28; meanwhile, mean SDS for weight was better, although below normal (−1.26 ± 0.24). In this study, perinatal growth data was not reported [41].

Other than the IFAR data on growth, there are another two studies that analyze growth parameters of FA patients. Anthropometric measurements from 45 patients with Fanconi anemia from the National Cancer Institute's inherited bone marrow failure syndromes (IBMFS) cohort were assessed. This cohort had a mean SDS for height of −2.1 ± 1.89. The height of over half of the participants (54%) was categorized as short (SDS > 2.0 SD); in this subset of patients, the mean SDS for height was −3.8 ± 1.5 [42]. Finally, the evaluation of 120 FA patients from the FA Comprehensive Care Clinic of Cincinnati Children's Hospital Medical Center revealed that median height SDS was shorter than expected in children and adults, irrespective of gender. Furthermore, this study presented birth information from 70 patients (59 children and 11 adults) from the cohort: 51% of the children and 3 adults were born small for gestational age; their median birth weight at term was 2.02 kg (range 1.5–2.6 kg) [43]. The intrauterine growth restriction seen in FA patients has also been documented in several case reports in which at-term birth weight ranges from 1,780 to 3,200 gr [44–49]. Even though growth abnormalities have been identified in FA patients with mutations in almost every* FANC* gene, it has been shown that patients with mutations in certain genes have a more severe growth delay. That is the case for patients with biallelic mutations in* FANCD1* [47, 48], as well as those who bear mutations in* FANCC* for whom an SDS for height of −3.84 has been found [41].

Endocrine abnormalities have been proven to be an inherent part of the FA phenotype; up to 80% of patients have one or more endocrine abnormalities. Besides growth, thyroid function and glucose homeostasis are frequently affected [40–43]. Considering the existing evidence for a relationship between low birth weight and an increased risk for noncommunicable adult diseases like diabetes, hypertension, heart disease, dyslipidemia, and osteoporosis [50], it is relevant to revise these diseases in FA. The FA cohorts in whom growth was assessed were also evaluated for endocrine status. Glucose homeostasis is affected in a large portion of FA patients; impaired glucose tolerance has been found in 27–68% of them, whereas diabetes has a prevalence of 8–10%. Dyslipidemia has been assessed in only a small portion of FA patients, but it has been found in 17–55% of the evaluated subjects [51]. There are conflicting results over bone mineral density (BMD) status in FA; the first time this was evaluated, 92% of the patients were found to have osteopenia or osteoporosis [42]; nevertheless, a study that evaluated a larger FA cohort found that low BMD is not a frequent finding [52]. Further research on this topic has raised the question of whether BMD deficit is intrinsic to the FA phenotype or a consequence of hematopoietic cell transplantation (HCT), an interrogation that is still awaiting to be answered [53]. Cardiovascular adult diseases are not relevant in the FA phenotype; heart disease in these patients is from the congenital type as 13% of patients have cardiac malformations [40], while hypertension has only been reported in two patients as the result of renal malformations [54].

Findings from the different FA cohorts show that short stature is an inherent feature of the FA phenotype, this feature does not respond to a single explanation; the apoptosis prone FA cellular phenotype is certainly a contributing factor for this. The fact that FA patients have a constitutionally defective pathway since conception could explain the clinical phenotype of RCIU. Short stature may also be exacerbated by accompanying endocrinopathies. The cooccurrence of many endocrine alterations has encouraged unifying explanations; some have proposed that endocrinopathies are secondary to increased cytokine activity found in FA cells [41], while others favor the view that some endocrine secretory cells might be damaged by high levels of reactive oxygen species which are known to be elevated in FA patients [43]. In line with the developmental origin of adult health and disease theory, it could also be possible that the increased prevalence of glucose homeostasis alterations and dyslipidemia reported in FA patients is a consequence of intrauterine growth restriction of these patients. Nevertheless, the attempted genotype-phenotype correlation of the endocrine phenotype in FA patients does not seem to support that. Patients with mutations in* FANCA* show a mild endocrine phenotype in which height is not severely affected and insulin resistance is mild, whereas mutations in* FANCC*, which are related to shorter stature, have the least insulin resistance [41]. Moreover, no direct relationship between birth weight and glucose tolerance was identified when it was intentionally looked for [43], although the number of patients in which these observations were made is limited.

### 7.3. Nijmegen Breakage Syndrome

**Figure 6:**
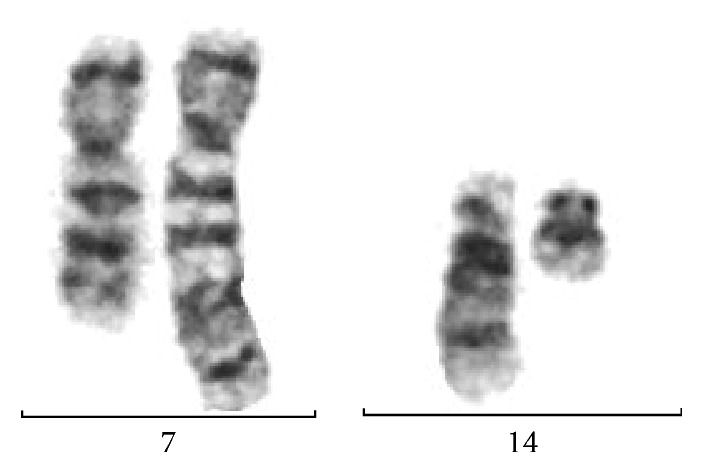
Translocation between chromosomes 7 and 14 is typical in NBS patients' cells.

Nijmegen breakage syndrome (NBS) is an autosomal recessive disease caused by biallelic mutations in* NBN*, a gene that encodes nibrin, a protein involved in DNA repair and cell cycle checkpoint regulation. It participates in the former by sensing double-strand breaks as part of the trimeric complex MRN, alongside MRE11 and RAD50. Meanwhile, for the latter, it contributes to the appropriate activation of ATM and ATR which are central transductors of the DNA damage response (DDR) [55]. The malfunction of nibrin translates in a cellular phenotype marked by chromosomal instability, radiosensitivity, reduced phosphorylation of ATM substrates, and S and G2/M cell cycle defects [56]. Chromosome instability is evidenced by cytogenetic methods in 10–60% of cells in the form of breaks and numeric and structural aberrations: translocations and inversions affecting chromosomes 7 and 14 are found in the majority of NBS patients and are considered a cytogenetic characteristic of this syndrome (Figure 6) [57].

The clinical impact of these alterations is a phenotype characterized by microcephaly, a distinctive facial appearance consisting of receding forehead and mandible and a prominent mid face with a long nose and philtrum, as well as immunodeficiency that leads to recurrent infections and an increased risk for the development of neoplasia, particularly leukemia and lymphoma. According to the international Nijmegen breakage syndrome study group, growth retardation is also a hallmark of this disease [58], although there is not many details about this feature in the literature.

Most of the information on the natural history of NBS available today comes from patients participating in registries, in which fairly large cohorts of NBS patients are included. There is a large representation of Slavic patients in these cohorts which correlates with the high carrier frequency of a founder mutation of* NBN* in this population. From these studies, it is evident that the most severely affected anthropometric measure in NBS patients is head circumference since all participants display microcephaly, even though only 75% display this feature at birth. When it comes to growth parameters, the same study states that all patients have growth retardation, which is described as proportionate and early occurring [58], although the specific temporality is not defined. A posterior report points out that the growth retardation seen in most NBS patients locates them in the 10th percentile in growth charts and that birth height and weight are usually within normal parameters [59]. Nevertheless, there are other authors who report prenatal and postnatal growth retardation [55, 60], and a tendency for short stature that is more evident during the first year of life [61]. Also, several case reports of NBS patients, in which birth weight is informed, demonstrate prenatal growth restriction [62–67], although this could represent a report bias. The more comprehensive data analysis on growth that includes perinatal information of NBS patients is the one that analyzes data from 67 patients followed for 15 years. Eighty percent of these patients were born at term, the girls were found to have a mean birth weight of 2.7 kg and mean birth height of 51.4 cm, while boys were reported to have a mean birth weight of 2.8 kg and mean birth height of 52.3 cm. Moreover, all patients, irrespective of gender, were found to have a mean height reduced by over 2 SDS at one year old [57]. These data support the fact that growth retardation in NBS is a prenatal phenomenon.

Nibrin has been found to have essential functions in the regulation of the cell cycle, which translates into an absolute need for this protein for cell proliferation [68]. It would seem logical that mutations of the* NBN* gene that compromise the appropriate function of nibrin would lead to abnormal growth when thought from an organism perspective. Mechanistically speaking, poor cellular growth caused by mutations in a particular gene can be thought of as the main explanation for the growth restricted phenotype, but it has been shown that mutations in other DDR genes that share the cellular phenotype of poor growth, like ATM, do not replicate the compromised growth phenotype at the organism level: growth retardation is absent in ataxia telangiectasia (AT). This data suggests that there are certainly other contributing factors to the abnormal growth phenotype seen in NBS patients [58].

### 7.4. Bloom Syndrome

Bloom syndrome (BS), is an autosomal recessive disorder caused by homozygous or compound heterozygous mutations in the* BLM *gene (15q26.1) that encodes the RecQL3 helicase.* BLM* has a critical role in the maintenance of genome stability acting at the interface between DNA replication, recombination, and repair [69, 70]. To date, over sixty different mutations, including nonsense and missense, have been identified; all of them result in the inactivation of* BLM* and the consequent loss of its helicase activity. The* BLM* gene encodes a 1,417 amino acid nuclear protein; its expression peaks during the S and G_2_/M phases of the cell cycle, which is consistent with its role in DNA replication and recombination.* BLM* specifically unwinds structures like forked DNA duplexes, RNA-DNA heteroduplexes, and R-Loops, which explains its importance during replication fork progression and transcription, besides it is central for Holliday Junctions (HJs) dissolution for HR. The dissolution of HJs by the topoisomerase III*α*-BLM complex cannot be replaced by any other RecQ helicase in the family [69, 70]. It has also been found to have a role in the annealing activity of the ssDNA, the proper sister chromatid segregation during mitosis, and telomere maintenance [70].

*BLM* can have both pro- and antirecombinogenic functions that are regulated by post/translational modifications, including phosphorylation, sumoylation, and ubiquitination [70].

*BLM* also enables sister chromatid segregation by processing unresolved replication intermediates that manifest in mitosis as ultrafine DNA bridges (UFBs) and, together with topoisomerase III*α*, RMI1, and RMI2, localizes to UFBs in anaphase. BML deficient cells exhibit and increase frequency of UFBs, suggesting a role of BLM in the resolution of these structures. Failure to resolve these UFBs leads to DNA breakage as mitosis proceeds [70].

**Figure 7:**
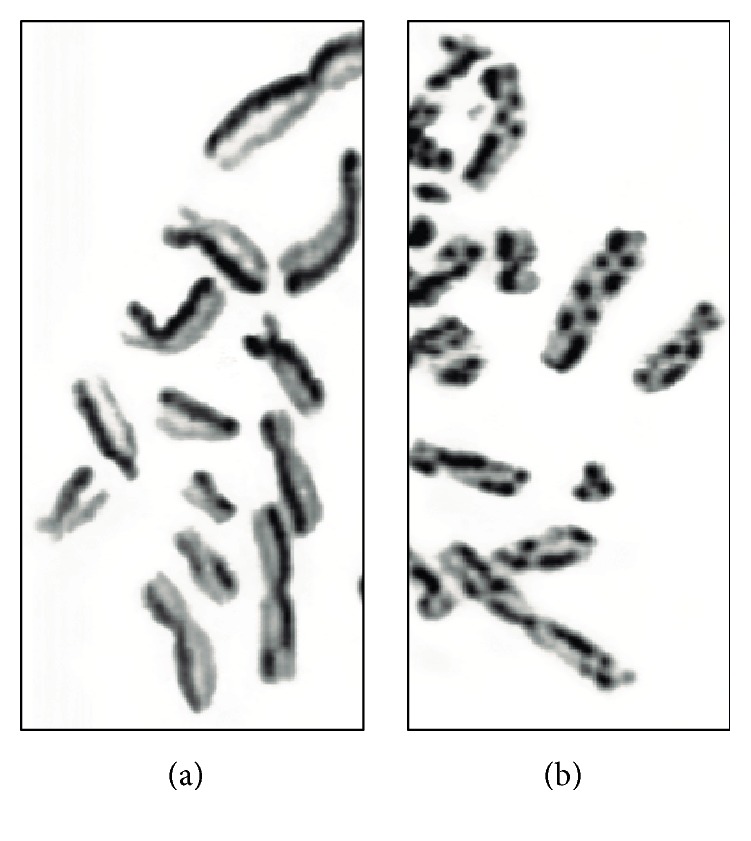
Sister chromatid exchange in cells from a healthy individual (a) and from a BLM patient (b).

Therefore, BS cells are characterized by an increase in chromosomal aberrations, including chromatid gaps and breaks, telomere associations, and quadriradial chromosomes resulting from unsolved recombination between homologous chromosomes. BS cells exhibit increased mutation rates, and the genomic instability includes elevated mitotic HR and unequal sister chromatid exchange (SCEs) (Figure 7). Perhaps the most characteristic feature is the over tenfold increase in SCEs, which results from crossover events during HR repair of damage replication forks [50, 69, 70].

Clinically, the more striking BS feature is IUGR; average birth weight at term is 1,850 g, and birth length is 44 cm. For both boys and girls, weight and length in BS are more than two standard deviations below normal, indicating that growth retardation has a prenatal origin. At postnatal ages, height remains below the normal range and is accompanied by a paucity of subcutaneous fat. Average adult height in males has been found to be 148 cm (130–162 cm) while in females it has been reported to be 139 cm (122–151 cm) [50, 70, 71].

**Figure 8:**
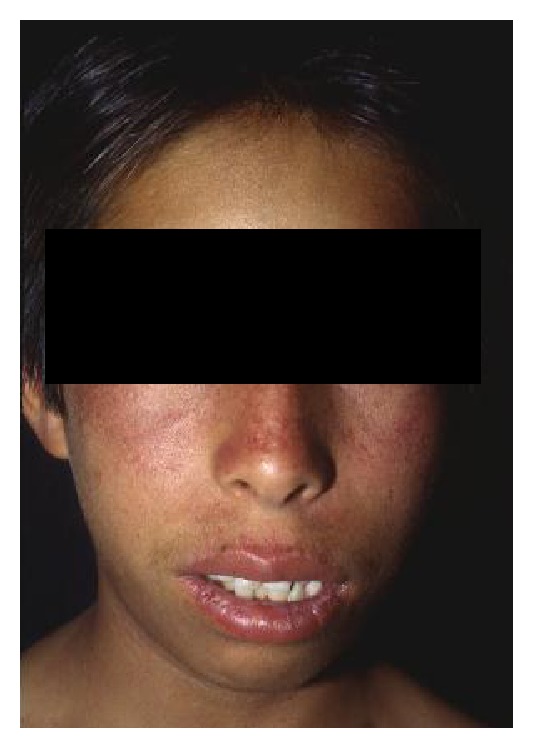
Bloom syndrome patient in whom the characteristic malar erythema is present.

BS patients have overall proportionated short stature but the head is reported to be small and narrow relatively to the body size; it is described as mild microcephaly and is accompanied by malar hypoplasia. Patients have been described to have a high pitched voice [50, 70]. The skin appears normal at birth, but in the first or second year of life, in response to sun exposure, children develop an erythematous rash with a butterfly distribution in the malar area and the back of hands and feet. The rash can include telangiectasia. The severity varies between patients; some can even lose their eyelashes and develop blistering around the mouth. The presence of café-au-lait spots and hypopigmented areas of skin associated with contiguous hyperpigmented areas is common (Figure 8). Intellectual abilities may be limited in some patients and normal in others [50, 70].

Other clinical conditions reported in BS patients are gastroesophageal reflux and diarrhea in infants; mild immunological deficiency with frequent episodes of otitis media; azoospermia or severe oligospermia in males and premature cessation of menstruation in females; minor anatomic defects; and increased risk for neoplasia, which is the main cause of dead in BS [70]. Another common medical condition in BS is diabetes mellitus associated with impaired glucose tolerance and insulin resistance; it has been reported in up to 16% of patients [70]. According to data from the Bloom's Syndrome Registry, diabetes in these patients tends to begin early with a mean age at diagnosis of 26.6 years. It is not associated with ketosis, and it does not have the classical hereditary pattern seen in type 2 diabetes [50, 72].

Short stature in BS is not due to hormonal causes; since the size of cells in persons with BS is normal, it has been hypothesized that there are fewer of them, due to either a decrease in the cell division rate, an increase in apoptosis, or even a combination of both. BS cells have trouble meeting the demands of fast cell divisions encountered in tissues during embryonic development. The leading hypothesis suggests that problems arising during DNA synthesis require a longer S phase to overcome the challenge, which translates into slowing of the cell division rate and an increase in apoptosis [70].

## 8. Concluding Remarks

This review has focused on discussing phenotypes of CIS in which there is a prenatal alteration of growth. The four syndromes discussed here are rare diseases whose prevalence is difficult to calculate since appropriate identification and diagnosis of affected individuals among the general population are not straightforward. It is important to note that the available information on the natural history of FA, NBS, and BS comes from the analysis of several patients that participate in registries. This strategy results in detailed information that permits a better analysis of the clinical phenotype of these rare diseases. Meanwhile, available data of SS is more anecdotic which results in a nonsystematic gathering of information, which is thus frequently incomplete.

**Figure 9:**
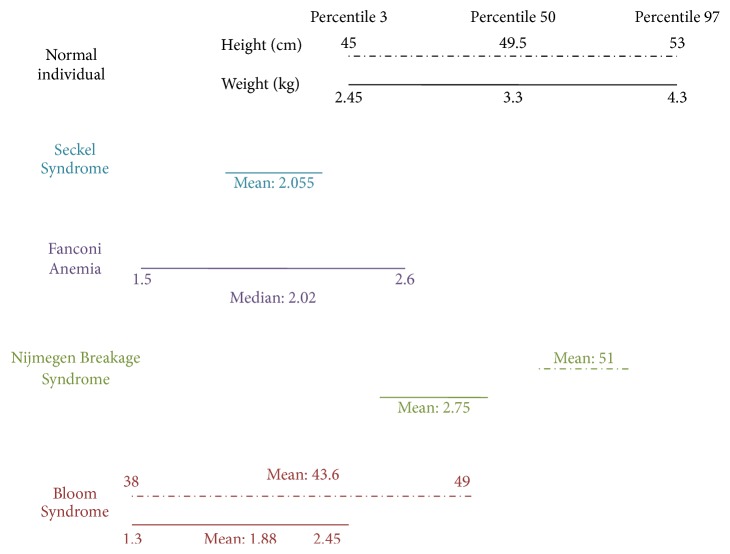
Anthropometric features at birth of chromosome instability syndromes with perinatal growth retardation. Comparison of the available growth data from SS, FA, NBS, and BS syndrome against birth data from the WHO's growth standards for infants. Although mean height and weight values of NBS patients are found to be within normal percentiles according to WHO standards; there are several authors who refer to prenatal growth restriction [55, 63, 65, 79].

From the available clinical data of these CIS, it is evident that growth is not affected in the same extent for all of these syndromes; the phenotypes that exhibit a more severely stagnated perinatal growth are BS and SS. The severe growth delay reported in these patients has been described as a pathognomonic sign of these diagnoses. In patients with FA, perinatal growth can also be severely affected, but not every FA patient shows this manifestation. Finally, NBS has been described to have growth retardation of prenatal origin of mild severity, almost within the lower normal range (Figure 9). The cephalic circumference can be affected in all of these syndromes; severe microcephaly is pathognomonic in SS and NBS, it is described as mild for BS patients and it is found in less than half of FA patients (Table 2).

When trying to understand why a subset of CIS shows prenatal growth retardation, it could be alleged that the intrinsic chromosomal instability is responsible for it. But this would not explain why prenatal growth retardation is not a universal CIS feature; for example, ataxia telangiectasia patients who have biallelic mutations in the ATM kinase gene do not show prenatal growth delay. It strikes that a molecular common feature shared by the prenatal growth retardation SIC phenotypes is that they are caused by mutations in genes that are necessary for an appropriate response to DSBs during the S phase of the cell cycle. The molecular defect in SS affects the functioning of ATR, the preferred DDR transducing kinase used by highly replicating ESCs. The FA/BRCA pathway, which is affected in patients with FA, detects ICLs upon replication fork arrest. Nibrin, the protein affected in patients with NBS, is induced during S phase for the detection of DSBs. And the BS helicase forms a FANCM-BLM complex needed to sense ICLs during S phase [73]. It could then be argued that altered DNA replication is an important factor for the development of intrauterine growth restriction in these CIS. Nevertheless, it must be kept in mind that the inherent chromosome instability found in these syndromes may also be affecting normal growth in the patients [27, 43, 57, 60, 71].

Being small for gestational age has been associated with a higher risk of noncommunicable adult diseases like glucose homeostasis disorders, dyslipidemia, hypertension, and others. It has been proposed that fetal reprogramming to enhance fetal survival, despite the risk for adult onset diseases, is an outcome that does not take into account the reason for the prenatal growth restraint [50, 72, 74]. It must be kept in mind that noncommunicable diseases are multifactorial entities in which genetic and environmental factors converge, and their cause cannot be attributed to a single explanation. However, when growth restriction is a key feature in syndromes with chromosome instability, the possibility that cell hypoplasia due to DNA repair malfunction is contributing to the growth restriction phenotype must be contemplated. Moreover, since none of these adult onset diseases is reported as an inherent element of the SS and NBS phenotypes, the direct connection between low birth weight and adult noncommunicable diseases is less of a straight shot. Furthermore, there are other Mendelian syndromes with mutations in DNA repair genes that characteristically show adult noncommunicable diseases but do not have IUGR; such is the case for Werner syndrome, a progeroid disease caused by biallelic mutations in another RecQ helicase (RecQ4) that clinically presents with postnatal short stature and a prevalence of type 2 diabetes mellitus of 70% [50, 70, 74].

Natural history of FA, NBS, and BS documented through the existing patient registries has allowed the identification of noncommunicable diseases to be a part of the clinical spectrum of these syndromes (Table 2). Meanwhile, from the information available through SS case reports, no adult onset disease has been found to be part of this phenotype. To the best of our knowledge, the direct investigation of nonevident endocrinologic alterations, like glucose homeostasis or dyslipidemia, has not been directly made in SS patients. For a rare disease like SS, such a study can only be done if a fairly large group of patients, like the one found in registries, is available. If such a study was to be performed, hidden features in SS patients could be unraveled.

Oxidative stress sensibility is a cellular feature shared by all the SIC syndromes with IUGR that could contribute to the explanation of the premature development of noncommunicable adult diseases in these patients. Reports have shown that BS and NBS cells have endogenous reactive oxygen species (ROS) overproduction and an impaired mitochondrial homeostasis [75]; it is also well known that FA cells show increased levels of oxidative damage [38, 76], and there is evidence that oxidative stress can activate ATR-mediated checkpoint signaling [77]. Since this kind of damage has soundly been related to age-associated diseases that result from tissue degeneration, exacerbated oxidative damage found in these syndromes could result in cellular aging that manifests as overall premature aging with particular symptoms like glucose homeostasis alterations [76, 77].

Fetal growth restriction is a multifactorial condition where wide arrays of factors converge. Fetal aspects, in particular those affecting the genetic constitution of the fetus, have been recognized to play an important role. It has been demonstrated that chromosomal abnormalities and monogenic syndromes caused by mutations in genes that participate in growth or metabolic pathways directly affect fetal growth [78]. Even though they are rare entities, CIS appear as another group of monogenic pathologies that broaden the list of genetic fetal factors of fetal growth restriction.

## Figures and Tables

**Table 1:** Chromosomal instability syndromes with intrauterine growth deficiency.

Syndrome	Genes	Function in DDR	Repair pathway	Cytogenetic alteration
Fanconi anemia	21 *FANC* genes	ICL detection and processing, generation of adducts and DSBs (sensors, mediators and effectors)	Homologous recombination	Chromatid and chromosomal breaks Radial figures

Seckel Syndrome 1	*ATR*	SSBs detection and signal transduction (transducer)	Homologous recombination Nonhomologous recombination	Chromatid and chromosomal breaks and Rearrangements

Nijmegen Breakage Syndrome	*NBN*	DSBs detection and signaling (sensor and mediator)	Homologous recombination Nonhomologous recombination	Chromatid and chromosomal breaks Aneuploidies Rearrangements affecting chromosomes 7 and 14

Bloom Syndrome	*BLM*	Helicase, process SSBs, and Holliday Junctions (effector)	Homologous recombination	Quadriradials Increase in sister chromatid exchange

**Table 2:** Growth features of chromosomal instability syndromes.

Syndrome “Loci”	At-term birth weight/height	Microcephaly	Adult height	Adult onset diseases
Seckel Syndrome “*ATR* 3q23”	Birth weight below −3 SD (2,055 g)	Severe −4 SD	Postnatal growth retardation (below −5 SD)	Not reported

Fanconi anemia “21 *FANC* genes”	Median weight: 2.02 kg (1.5–2.6 kg)	In 20–50%	50–60% of patients with median height <−1.8 SDS Women: −3.4 SDS Men: −4.4 SDS	Cancer Diabetes mellitus Dyslipidemia Altered bone mineral density

Nijmegen Breakage Syndrome “*NBN* 8q21”	Mean weight/height girls 2.7 kg/51.4 cm Mean weight/height boys 2.8 kg/52.3 cm	100%	Mean height for women −1.8 SDS Mean height for men −2.3	Cancer

Bloom Syndrome “*BLM* 15q26.1”	Mean weight 1.89 kg ± 0.35 kg for boys Mean weight 1.87 kg ± 0.35 kg for girls Mean height 43.4 cm ± 4.4 cm for boys Mean height 43.8 cm ± 2.8 cm for girls	Mild microcephaly	Mean height 145.5 cm ± 7.6 cm males Mean height 141.5 cm ± 6.1 cm for females	Cancer Diabetes mellitus

See [27, 33, 40, 43, 50, 57, 69].
